# GAD antibodies in neurological disease: a critical evaluation of the utility and treatment implications of GAD antibodies in clinical practice

**DOI:** 10.1007/s00415-025-12926-3

**Published:** 2025-02-22

**Authors:** Rachel L. Brown, Gilbert Thomas-Black, Hector Garcia-Moreno, Michael Chou, Zofia Fleszar, Michael S. Zandi, Miles Chapman, Andrew J. Church, Melanie Hart, Paola Giunti, Angela Vincent, Michael P. Lunn

**Affiliations:** 1 National Hospital for Neurology and Neurosurgery, University College London Hospitals NHS Foundation Trust, London, WC1N 3BG UK; 2 Department of Neuromuscular Diseases, UCL Queen Square Institute of Neurology, University College London, London, WC1N 3BG UK; 3 UCL Institute of Immunity and Transplantation, University College London, London, NW3 2PP UK; 4 Ataxia Centre, Department of Clinical and Movement Neurosciences, UCL Queen Square Institute of Neurology, London, WC1N 3BG UK; 5 Department of Neurodegenerative Diseases, Center for Neurology and Hertie Institute for Clinical Brain Research, University of Tübingen, 72076 Tübingen, Germany; 6 Department of Neuroinflammation, UCL Queen Square Institute of Neurology, University College London, London, WC1N 3BG UK; 7 Nuffield Department of Clinical Neurosciences, John Radcliffe Hospital, University of Oxford, Oxford, OX3 9DU UK; 8 Centre for Neuromuscular Disease, National Hospital for Neurology and Neurosurgery, Queen Square, London, WC1N 3BG UK

**Keywords:** Glutamic acid decarboxylase, Antibody, Stiff person syndrome, Cerebellar ataxia, Limbic encephalitis, Epilepsy

## Abstract

**Background:**

The interpretation of antibodies to glutamic acid decarboxylase 65 (GAD-Abs) in neurological practice is challenging. GAD-Abs are not considered directly pathogenic and immunotherapy guidelines are lacking.

**Methods:**

We undertook a single-center retrospective service evaluation of GAD-Abs, documenting clinical features, immunotherapy responses, and outcomes of 335 patients with positive GAD-Abs measured by indirect ELISA between 2012 and 2020. The serum:CSF ratio of GAD-Ab values was used as a surrogate for intrathecal synthesis.

**Results:**

168 (50%) patients had diagnosed neurological disorders (GAD-ND). Ninety-six had neurological disorders often or sometimes associated with GAD-Abs, i.e., stiff person syndrome spectrum disorders (SPS-SD, *n* = 26), cerebellar ataxia (*n* = 21), epilepsy (*n* = 19), encephalitis (*n* = 18), or any combination of these (“mixed”, *n* = 12). Seventy-two had other neurological disorders (ONDs) not typically associated with GAD-Abs.

We defined a cut-off of 10,000 IU/mL a priori and a posteriori for GAD-Ab associated NDs, but identified values > 10,000 IU/mL in 21% and 11% of patients with ONDs or diabetes respectively, and < 10,000 IU/mL in 39% patients with classical GAD-Ab syndromes, indicating low assay specificity and sensitivity.

Low serum: CSF GAD-Ab ratios were consistent with intrathecal synthesis in 12/19 tested; 25/54 patients had oligoclonal bands. 30/50 patients given adequate immunotherapies had partial (*n* = 17) or good (*n* = 13) responses, particularly those with SPS-SD or limbic encephalitis. Within the limitations of small subgroups and routine laboratory titrations, patients with GAD-Ab values > 10,000 IU/mL, intrathecal synthesis of GAD-Abs, or oligoclonal bands, were not more likely to improve with immunotherapies than those with GAD-Ab values < 10,000 IU/mL and a non-inflammatory CSF. Rather, treatment response correlated with disease group, principally SPS-SD and encephalitis.

**Conclusions:**

These results suggest caution in over-interpreting GAD-Abs values. Better biomarkers for identifying patients with immunotherapy responsive GAD-Ab disease are needed.

## Introduction

Glutamic acid decarboxylase (GAD) is the rate-limiting enzyme in the catalysis of the excitatory neurotransmitter glutamic acid to inhibitory gamma-aminobutyric acid (GABA), and found primarily within GABAergic neurons and pancreatic β-cells [1, 2]. Autoantibodies to GAD65 (GAD-Abs) occur in type 1 diabetes (T1DM), latent autoimmune diabetes in adults (LADA) and autoimmune polyendocrine syndrome type 1 (APS1) as a marker of pancreatic destruction [3–6]. Traditionally very high GAD65-Ab values were found in patients with stiff person spectrum disorders (SPS-SD) and became part of their definition, but similar levels were identified in case series of cerebellar ataxia (CA), temporal lobe epilepsy, limbic encephalitis or combinations of these (‘mixed’ presentations) [7–10]. Given the intracellular location of GAD, the relevance and function of GAD-Abs across these disorders remains uncertain.

GAD-Ab assays are designed for diabetes, rather than specifically identifying possible immune-mediated neurological disorders. Originally, radioimmunoprecipitation assays (RIA) suggested that GAD-Abs only > 2000 U/mL were likely to be neurologically relevant, but RIA has been superseded by non-radioactive ELISAs with units as International Units (IU)/mL [8, 11]. A recent study nominated > 10,000 IU/mL as a suggested ELISA cut-off for GAD-Ab related neurological disorders [11].

GAD-Abs have been assayed at the Institute of Neurology for more than 30 years and the current ELISA for more than 10 years. The requests for serum and sometimes CSF GAD-Abs are made in SPS-SD but also in CA, epilepsy and encephalitis patients. Many GAD-Ab positive patients have undergone one or more immunotherapy treatments. We retrospectively reviewed the ‘real-life’ results and sequelae of positive GAD-Ab tests, considered a threshold for identifying GAD-Ab neurological conditions, attempted to quantify the immunotherapy responses, and examined possible factors predicting good response.

## Materials and methods

### Ethics

The study was approved by the Quality and Safety Team at the National Hospital for Neurology and Neurosurgery and University College London Hospitals (registration number 90-202021-SE), as a service evaluation of the utilization and impact of GAD antibody testing.

### Patients

All patients with serum tested for GAD-Abs at the Neuroimmunology and CSF Laboratory (NICL) at the National Hospital for Neurology and Neurosurgery (NHNN) between the 2012 and 2020 were identified through laboratory records. Patients referred from within University College London Hospitals (UCLH) NHS Foundation Trust, for whom electronic clinical records were available, were included in the analysis. All patients who had serum tested for GAD-Abs for neurological indications had undergone neurological assessment at NHNN. Demographics, paraclinical data, treatment histories and outcomes were extracted from patient records. Final diagnoses were either diabetes and/or those given by the treating consultant neurologist, agreed independently by two authors (RB and MPL) according to published criteria [12, 13]. For SPS-SD, ‘classic’, ‘partial’ and ‘SPS-plus’ were grouped singly as ‘SPS-SD’. SPS/PERM was identified separately. The patients allocated to the cerebellar ataxia group had no features of SPS-SD. Patients with clinical features including both ataxia and SPS-SD were allocated to the "mixed" group.

### GAD-Ab assay

All sera and CSF (when available) were tested in duplicate for GAD-Abs by indirect ELISA (Euroimmun, Lübeck, Germany). Sera and CSF were first tested undiluted with serum positivity defined as > 10 IU/mL, according to the manufacturer’s instructions. A specific cut-off for CSF was not provided, but based on in-house validation where normal and disease controls are 0 IU/mL, any positive value (i.e., > 0 IU/mL) was used here. The results were calculated automatically using plate reader software at both 405 and 450 nm as per manufacturer instructions. For results greater than 35 IU/mL, results from the 405 nm curve were used. For results less than 35 IU/mL, results from the 450 nm curve were used. Sera or CSFs > 2000 IU/ml were assayed at 1:50 and 1:500 to identify accurate values (reported as IU/mL after correction for the relevant dilution factor).

### Statistical analysis

For analysis of serum and CSF GAD-Abs values, the earliest available result or the first with a titrated value was used. For graphing, values over 1,000,000 IU/mL were capped. A CSF GAD-Ab index was calculated by using the ratio between serum and the nearest proximate CSF value if an exactly contemporaneous serum value was not available. Mann–Whitney *U* non-parametric tests compared GAD-Ab values between groups, Chi^2^ and Fisher’s exact test compared responses to treatment dependent upon clinical or laboratory characteristics. Analyses and graphical data were generated using GraphPad Prism, Version 9.2.

### Responses to treatment

Electronic records were reviewed for treatments and documented responses. Immunotherapy regimes and documented responses were recorded (RB) and checked pragmatically for appropriateness and dose adequacy (MPL). Treatment considered ‘adequate to demonstrate responsiveness’ included sufficient neurological immunosuppression doses of at least one agent for a sufficient time period (e.g., IVIG given for at least two courses of 2 g/kg, PLEX at least two treatments consisting of 3–5 days whole body exchanges, a steroid-receptor saturating dose of corticosteroid [1 mg/kg prednisolone for at least 1 month or equivalent], or a treatment dose of a steroid sparing agent for at least 6 months).

Treatment responses were independently and blindly scored by RB and MPL. In discrepant cases, differences were resolved by discussion. Degree of clinical improvement in those patients given adequate immunotherapies was scored as 0 = no improvement, 1 = partial improvement or 2 = substantial (good) improvement. Where possible scores were based on functional outcome measures related to disability such as the 10-m walk test (10MWT), a relevant disease specific score (e.g., Scale for the Assessment of Ataxia), or other disease-specific measure (e.g., seizure frequency). For encephalitis, SPS-SD and mixed cohorts, a retrospective mRS was reconstructed for pre- and post-treatment clinical descriptions and functional outcomes. In general, either an improvement of > 1 mRS point, or mRS improvement to 1 or 0 was considered a ‘good response’; these data were combined with a qualitative interpretation of heterogeneous outcome measures and other relevant treatment factors.

### Data availability

Data are available on reasonable request to the corresponding author.

## Results

### Overall cohort

**Fig. 1:**
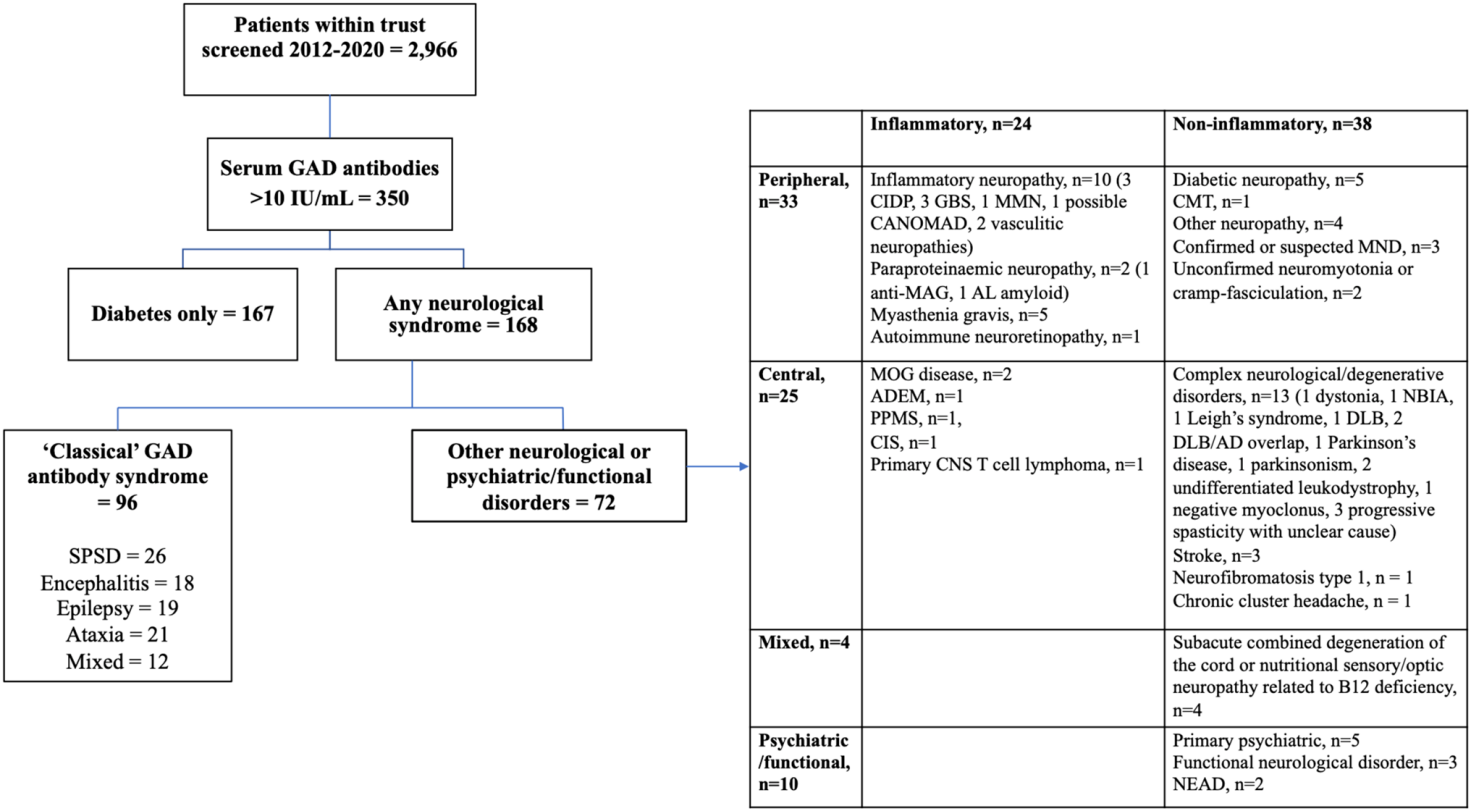
Summary of overall cohort

5959 valid requests for serum GAD-Ab testing were sent to NIC between 2012 and 2020. Of these, 3748 were from UCLH NHS Foundation Trust representing 2966 patients. 350 (11.8%) had serum GAD-Abs above the manufacturer’s cut-off (10 IU/mL) (Fig. 1). 15 patients with insufficient clinical data were excluded from the study. 25 patients (14 ‘diabetes only’, 11 neurological diseases) were excluded from quantitative analysis as their GAD-Ab values were recorded as > 2000 IU/mL (i.e., the top standard of the ELISA assay) and not titrated.

### GAD-Ab values and cut-off for neurological conditions

**Fig. 2:**
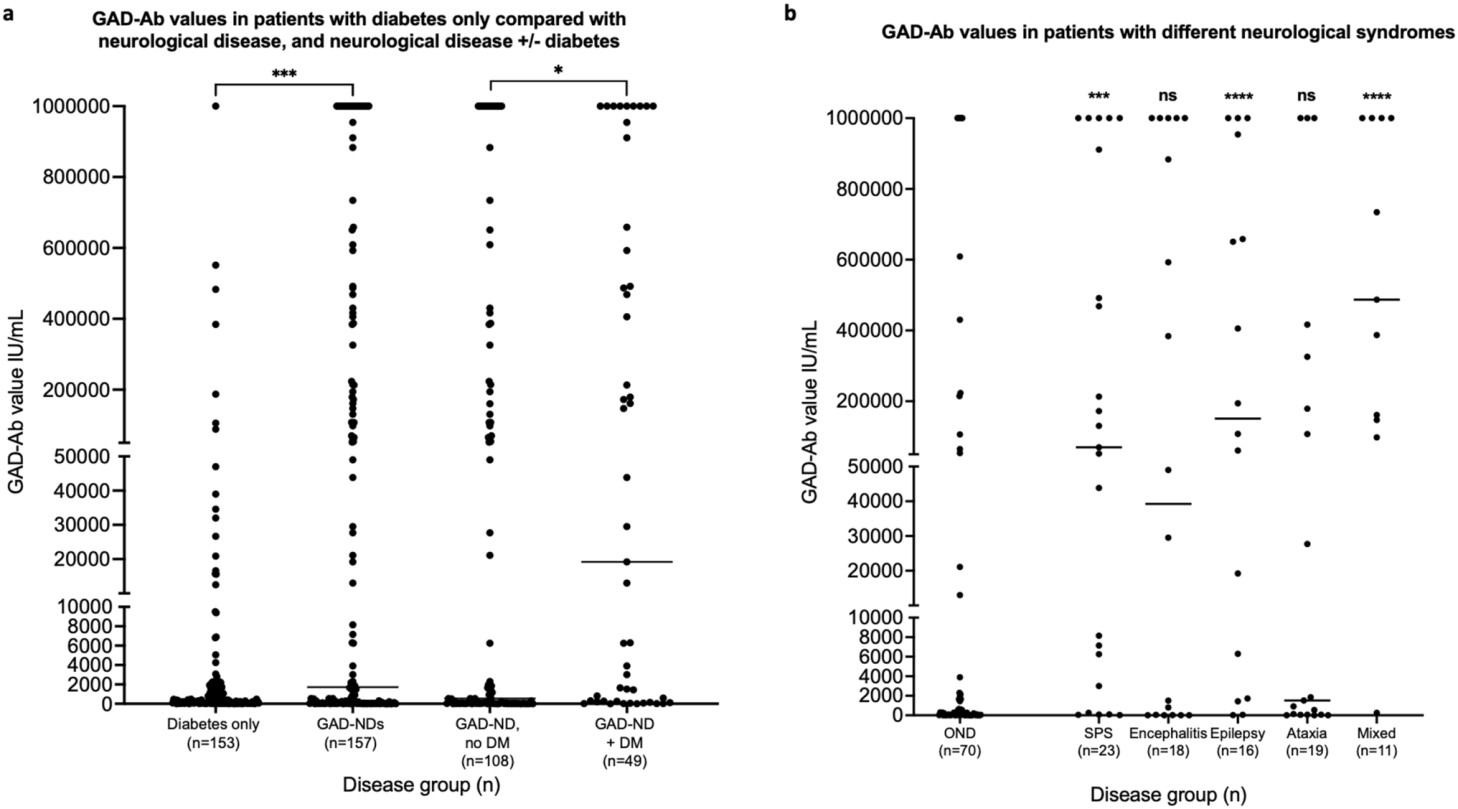
GAD antibody values in different disease groups. GAD-Ab values were higher in patients with GAD-Abs and neurological diseases (GAD-NDs) compared to diabetes only, and in patients with GAD-NDs and diabetes compared with GAD-ND only (**a**). Patients with SPS, epilepsy or mixed syndromes had significantly higher GAD-Ab values than patients with non-classical syndromes (OND); those from patients with CA were not significantly different to OND (**b**). *n* = number of patients; bars indicate median values;* indicates *p* ≤ 0.05, ***p ≤ 0.001, ****p ≤ 0.0001, ns = *p* > 0.05

Figure 2a shows the GAD-Ab values of patients with diabetes only (*n* = 153) and compares with all those (*n* = 157) with one or more GAD-Ab associated neurological diseases/diagnoses (NDs). Patients with GAD-Ab and neurological diseases (GAD-NDs) had higher GAD-Abs values (*p* = 0.0006) than those with diabetes only, but many of the GAD-ND values were within the diabetic range. GAD-Ab values in the 49 GAD-NDs with concomitant diabetes (43/49 type 1 or LADA) were higher than those who did not have diabetes (*p* = 0.0115, Fig. 2a).

Figure 2b shows the serum GAD-Ab values of 70 ONDs compared with the ‘classical syndromes’ (23 SPS-SD, 18 autoimmune encephalitis, 16 epilepsy, 19 CA, and 11 mixed). In all groups (with exception of the mixed syndromes, where all but one value was > 10,000 IU/mL) there was a wide range of values. Only patients with SPS-SD, epilepsy, and mixed syndromes showed significantly higher serum values than the ONDs. However, the encephalitis group could be divided between those with otherwise seronegative LE and high GAD-Ab values (all but one > 10,000 IU/mL), and those with other encephalitis syndromes (e.g. LGI1-Ab or Rasmussen’s) whose GAD-Ab were all < 2000 IU/mL.

Using an ELISA cut-off value of 10,000 IU/mL for neurological disease as previously suggested (11), 17/153 (11%) of ‘diabetes only’ and 15/70 (21%) OND patients would be included in the ‘relevant neurological disease’ group whilst 34/87 (39%) patients with ‘classical’ GAD-Ab related syndromes were excluded. Therefore, this previously proposed cut off was neither specific nor sensitive for assessing the value of GAD-Ab values in our cohort.

**Fig. 3:**
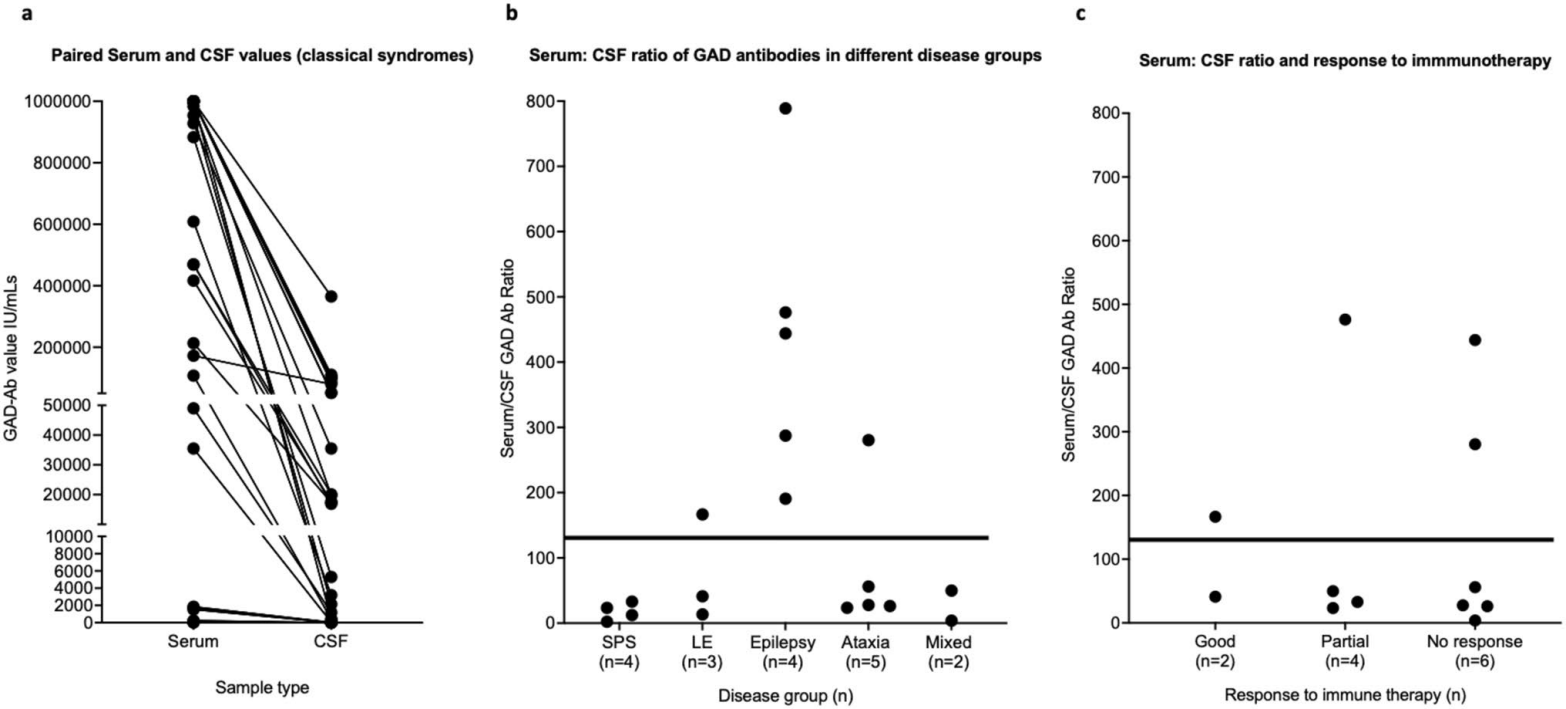
Serum and CSF GAD antibody profiles. **a** Shows paired serum and CSF GAD antibody values in all patients in whom this was available, and **b** GAD-Ab ratios in different disease groups for all patients treated or untreated with immunosuppression. **c** GAD-Ab ratios and responses in patients deemed to have been given adequate immunotherapy to determine treatment responsiveness. Line indicates level below which intrathecal synthesis of antibodies is likely

Available paired serum and CSF values are shown in Fig. 3a. Normal serum IgG varies between 6 and 16 mg/mL and CSF IgG between 0 and 0.045 mg/mL (often given as 0–4.5 mg/dL); thus the range of normal serum/CSF IgG ratios can vary between 133 (6/0.045) to 1600 or even greater. When applied to specific antibodies, values below 133 imply an excess of intrathecally synthesized antibody. CSF GAD-Abs were not detectable in six patients (3 SPS-SD, 2 CA, 1 encephalitis) with low serum values (< 2000 IU/mL). Of those with detectable CSF GAD-Abs (values between 6 and 365,111 IU/mL) and a paired serum sample, ratios for serum/CSF GAD-Abs ranged from 2.17 to 788.89, with a clear distinction between those > 133 (166–788) and those < 133 (2–56) (Fig. 3b). Notably, 4/4 patients with SPS-SD, 2/2 with mixed syndromes, 4/5 with CA and 2/3 with limbic encephalitis patients had evidence of intrathecal GAD-Ab synthesis whereas, despite the high serum values, all five epilepsy patients had ratios within the normal range.

### Disease groups and treatment outcomes

Clinical features of GAD-ND groups are summarized in Table 1. The treatment responses of individual patients are summarized in Table 2. This was a retrospective study and, as such, treatment regimes, outcomes and their timings were not standardized across the cohort, but all patients were assessed by experienced neurologists at our center.

**Table 1:** Summary of clinical features of patients with GAD-Abs and neurological syndromes

	SPS-SD (*n* = 26)	Encephalitis (*n* = 18)	Epilepsy (*n* = 19)	Ataxia (*n* = 21)	Mixed (*n* = 12)	Other neurological disorders (*n* = 72)
Median age at onset, years (range)	50 (23–78)	31 (13–82)	25 (7–65)	60 (12–76)	38 (16–61)	49 (16–78)
Female, *n* (%)	16 (62)	10 (56)	15 (79)	13 (62)	10 (83)	43 (60)
Time to GAD ab testing < 1 year	12 (46)	15 (83)	2 (11)	9 (43)	3 (25)	23 (32)
Other AI diseases, *n* (%)	18 (69)	7 (39)	14 (74)	13 (62)	8 (67)	29 (40)
T1DM or LADA (IDDM)	12 (46)	5 (28)	9 (47)	3 (14)	6 (50)	18 (25)
Autoimmune thyroid disease	12 (46)	3 (17)	8 (42)	7 (33)	6 (50)	5 (7)
> 1 autoimmune disease diagnosed	11 (42)	1 (6)	9 (47)	3 (14)	5 (42)	9 (13)
Other neuronal or thyroid antibodies (*n*)	GlyR 4, amphiphysin 1, Sox1 2, Zic4 1, VGKC 1, thyroid 5	GlyR 1, LGI1 2, VGKC 1, GABA_B_R 1, thyroid 3	VGKC 1, VGCC 1 (CSF), thyroid 3	AChR 1, thyroid 1	GlyR 3, Sox1 1, Zic4 1, thyroid 3	PND 5, AChR 3, MuSK 2, Gangliosides 2, MAG 1, Neurofascin 155 1, GlyR 1, MOG 2, thyroid 4
History of malignancy, *n* (%)	5 (19)	4 (22)	0 (0)	2 (10)	1 (8)	12 (17)

AChR: acetylcholine receptor; AI: autoimmune; GABA_B_R: gamma-aminobutyric acid type B receptor; GlyR: glycine receptor; IDDM: insulin dependent diabetes mellitus; LADA: latent autoimmune diabetes of adults; LGI1: leucine rich glioma inactivated 1; MAG: myelin associated glycoprotein; MOG: myelin oligodendrocyte glycoprotein; MuSK: muscle specific kinase; PND: paraneoplastic disease; SPS-SD: stiff person syndrome spectrum disorders; T1DM: type 1 diabetes mellitus; VGCC: voltage gated calcium channel; VGKC: voltage gated potassium channel

VGKC antibodies measured prior to development of assays for antibodies to LGI1 and CASPR2

**Table 2:** Response to immunotherapy in patients deemed to have had an adequate trial of treatment

Condition	Age of Onset (decade)	Unmatched OCB (yes/no); CSF lymphocytes	Serum values (IU/mL)	Serum/CSF values (surrogate for ITS)	Other antibody	Time from onset to first immunotherapy (years)	Treatment given	Best treatment outcome (pre to post treatment)	Response
SPS + Ep	6th	No; WCC < 5	387,066		Glycine R	1	PLEX, rituximab	mRS 2 > mRS 1(SPS) (epilepsy responded to anti-seizure medication)	Good
SPS	5th	Yes	> 2000		–	1	PLEX × 1 course then regular IVIG	mRS 4 > mRS 2	Good
SPS	5th		69,822		–	4	Regular IVIG	mRS 3 > mRS 1	Good
SPS	7th		> 1,000,000		–	2.5	PLEX × 3 courses, PLEX/IVIG × 2 courses, IVIG	mRS 2 > mRS 1	Good
SPS	3rd	No; WCC 7	71		–	1	PLEX × 2 courses, IVIG, rituximab, MMF	mRS 2 > mRS 0	Good
SPS	6th		252		Glycine R	4	Regular PLEX; rituximab; cyclophosphamide	mRS 3 > mRS 1	Good
LE	2nd	No; WCC < 5	49,000	41.14	–	0	Steroids	mRS 5 > mRS 1	Good
LE	3rd	WCC 8	883,500	166.69	–	0	Steroids; PLEX; rituximab	mRS 5 > mRS 0	Good
LE	3rd	Yes; WCC < 5	> 1,000,000		Low titer GABA_B_R	0	PLEX; IVIG	mRS 3 > mRS 1	Good
LE	3rd	Yes; WCC 9	> 1,000,000		–	0	Steroids; PLEX; azathioprine	mRS 3 > mRS 2	Good
LE	4th	WCC < 5	17		–	0.25	Steroids; IVIG	mRS 5 > mRS 2	Good
Epilepsy	1st	No	1440		–	10	Steroids (3 days IVMP); PLEX × 1 course; then regular IVIG	75% seizure reduction	Good
A + Ep	6th		98,000		–	1	Steroids; PLEX; MMF; cyclophosphamide	mRS 2 > mRS 1	Good
A + Ep	6th	No; WCC < 5	> 1,000,000		–	2	IVIG × 2 courses	mRS 3 > mRS 2 (SARA 12.5/40 > 9/40)	Partial
SPS + Ep	3rd	Yes; WCC < 5	> 2000		–	1	IVIG	mRS 1 > mRS 1 (modest improvement in 10 m walk)	Partial
SPS + A + Ep	4th	Yes; WCC < 5	984,035	49.95	Glycine R	9	PLEX × 2 courses and IVIG × 2 courses	mRS 2 > mRS 1 (SPS) (epilepsy improved with anti-seizure medications)	Partial
SPS/PERM	6th	Yes; WCC 12	> 1,000,000		–	1	Steroids; IVIG; PLEX; MMF	mRS 5 > mRS 4	Partial
SPS	6th	Yes; WCC < 5	1,712,416	32.94	–	1.5	Regular IVIG	mRS 4 > mRS 4 (improvement in stiffness and spasms)	Partial
SPS	3rd	No; WCC < 5	468,500	23.30	–	6	Regular IVIG	mRS 4 > mRS 3	Partial
SPS	5th		130,705		–	1	Steroids; azathioprine; PLEX	mRS 3 > mRS 2	Partial
SPS	6th	Yes; WCC < 5	> 2,000,000		–	1	Steroids then regular IVIG	mRS 3 > mRS 3	Partial
SPS	4th	Yes; WCC < 5	1,488,210		–	1	Steroids; IVIG; PLEX; rituximab		Partial
SPS	5th	No; WCC < 5	7156		–	1	PLEX × 5 courses; rituximab; regular IVIG	mS 2 > mRS 1	Partial
SPS	6th		491,636		–		Regular IVIG	mRS 4 > mRS 3	Partial
SPS	7th		52,000		–	4	IVIG × 2 courses	mRS 3 > mRS 2	Partial
SPS	8th		6256		Glycine R	1	Steroids; PLEX; rituximab; regular IVIG	mRS 3 > mRS 2	Partial
LE	4th	Yes; WCC < 5	> 1,000,000		–	2	Steroids	mRS 2 > mRS 1	Partial
LE	6th	No; WCC 10	> 1,000,000		–	0	Steroids/IVIG	mRS 5 > mRS 4	Partial
Epilepsy	2nd	No; WCC < 5	194,266	476.14	–	5	PLEX × 2 courses then azathioprine	Reduction in seizure frequency following PLEX with wearing off effect	Partial
Ataxia	5th	Yes; WCC < 5	9		–	0	Steroids, IVIG	OMS—improvement on unified myoclonus rating scale, stabilization of ataxia	Partial
SPS + A + Ep	4th	Yes; WCC < 5	> 1,000,000		–	1	Steroids; PLEX; IVIG	mRS 3 > mRS 5	None
SPS + A	5th	Yes; WCC < 5	1,382,086	3.78	–	3	IVMP; prednisolone; PLEX × 2 courses; IVIG × 3 courses; azathioprine	mRS 3 > mRS 4 (SARA 7.5/40 > 25/40)	None
SPS + A	4th	Yes; WCC 6	> 1,000,000		Glycine R	3	IVMP; PLEX × 2 courses; IVIG	mRS 3 > mRS 3	None
SPS + Ep	3rd		161,082		Weak positive Sox1, previous Zic4	19	IVIG × 4 courses	mRS 2 > mRS 2 (seizures controlled following epilepsy surgery)	None
A + Ep	5th		734,237		–	10	IVIG × 3 courses; MMF	mRS 3 > . mRS 4	None
SPS/PERM	6th	No; WCC < 5	16		Weak positive Sox1 and Zic4	1	Steroids; IVIG; PLEX; rituximab	mRS 2 > mRS 3	None
SPS/PERM	5th	WCC < 5	51		–	0	Steroids; PLEX	mRS 5 > mRS 6	None
SPS	3rd	Yes; WCC < 5	> 2000		–	3	PLEX × 1 course; IVIG × 1 course	mRS 4 > mRS 4	None
SPS	5th		8150		–	8	IVIG and PLEX × 3 courses	mRS 2 > mRS 2	None
LE	2nd		29,500			0	PLEX, IVIG × 3, rituximab, alemtuzumab, mycophenolate	mRS 5 > mRS 4	None
Epilepsy	3rd	No; WCC < 5	954,028	443.94	VGKC (very low positive, later negative)	2	Prednisolone 60 mg for 2 months; IVIG (single course)	No change	None
Epilepsy	3rd		650,790		–	30	PLEX × 6 courses	No change	None
Ataxia	5th	Yes; WCC < 5	928,000	26.14	–	1	PLEX, cyclophosphamide	SARA 6/40 > 10/40	None
Ataxia	8th	Yes; WCC < 5	469,917	27.71	–	3	PLEX, cyclophosphamide		None
Ataxia	7th	No; WCC < 5	1850	56.06	–	1	PLEX × 3 courses then IVIG	SARA uk > 22/40	None
Ataxia	2nd	No; WCC < 5	1521		–	6	PLEX; mycophenolate	SARA 6.5/40 > no response	None
Ataxia	7th	WCC < 5	107,400	280.42	–		Steroids, PLEX, IVIG, azathioprine	SARA uk > 9.5/40	None
Ataxia	5th	Yes; WCC < 5	> 1,000,000		–	0.75	Steroids 4 months	SARA 4.5/40 > no response	None
Ataxia	5th	WCC < 5	65		–	4	IVIG × 3 courses		None
Ataxia	8th	Yes; WCC < 5	> 2000		–	5	Steroids, PLEX, IVIG, azathioprine	SARA 15.5/40 > SARA 20/40	None

Empty cells represent insufficient evidence or not done

OCB: oligoclonal bands; CSF: cerebrospinal fluid; ITS: intrathecal synthesis; SPS: stiff person syndrome spectrum disorders; Ep: epilepsy; LE: limbic encephalitis; A: ataxia; PERM: progressive encephalomyelitis with rigidity and myoclonus; OMS: opsoclonus myoclonus syndrome; mRS: modified Rankin score; SARA: scale for the assessment and rating of ataxia; uk: unknown; WCC: white cell count (WCC < 5 considered normal)

#### Stiff person spectrum disorders group

Twenty-six patients had typical GAD-Ab associated SPS-SD, including four (15%) with progressive encephalomyelitis with rigidity and myoclonus (PERM). Neurophysiological examinations corroborated the clinical diagnosis in 11 of 21 with studies available. Twenty-three (88%) SPS-SD patients received one (*n* = 9, 35%) or more (*n* = 14, 54%) forms of immunotherapy, often over years. IVIG was most common (*n* = 20, 77%), including eight regularly dosed over ≥ 1 year. Fourteen (54%) patients received one or more courses of plasma exchange (PLEX), three regularly over at least one year (one with IVIG given after each PLEX). Of the 19 patients who were thought to have had an adequate trial of treatment and who had follow-up data available, 15 (79%) reported benefit varying from moderate and temporary to significant and lasting improvement in mobility and independence. Overall, immunotherapy was beneficial but required on-going maintenance in most of patients.

#### Encephalitis group

18 patients had clinical features meeting Graus criteria for encephalitis [13]. Six required ICU admission and most required inpatient ward admission. Median time to GAD antibody testing was short (11/18 tested in the initial illness) reflecting the acute presentation. Seven (39%) patients were diagnosed with specific encephalitis syndromes, including two with LGI1-Ab encephalitis who were treated successfully within current guidelines and made good recoveries.

Of the 11 without an alternative diagnosis, all had subacute onset limbic encephalitis (LE), mostly with high GAD-Ab values (all but one > 10,000 IU/mL), and commonly seizures. All 11 had abnormal MR imaging, involving the hippocampi or medial temporal lobe structures in 10 (9 abnormal T2 or FLAIR signal, one hippocampal sclerosis). Immunotherapy was tried in 10/11 (one patient had seizure remission following temporal lobectomy and was not treated with immunotherapy). One patient was given a trial of treatment for post-encephalitis epilepsy without benefit (treatment details for initial illness unknown). One was lost to follow up. Of the 8 patients treated in the acute phase and with follow up, 7 had a good or partial recovery, whilst 1 had refractory epilepsy, physical and cognitive disability despite adequate treatment.

#### Epilepsy group

Nineteen patients had epilepsy without fulfilling criteria of encephalitis. Most (16/19) had longstanding focal epilepsy, including 13/16 temporal lobe epilepsy, and 14/16 with treatment resistant disease (defined as ongoing seizures despite two or more appropriate and tolerated anti-epileptic drug [AED] regimens). The remaining three patients had late onset symptomatic seizures (*n* = 2) without evidence of encephalitis, or an inherited generalized epilepsy syndrome (*n* = 1).

Six (38%) of the 16 patients with treatment-resistant focal epilepsy (all with > 5 prior AEDs) underwent trials of immunotherapy (only 1 within 2 years of onset). Three received first line therapy only (2 PLEX, 1 steroids and IVIG), two received first line therapy (PLEX alone or steroids, PLEX and IVIG) followed by azathioprine and one received azathioprine alone. Three of six patients showed some improvement. Three had reduction in seizure frequency after one and two cycles of PLEX leading to immunotherapy continuation. Of these, one was commenced on azathioprine and one had further PLEX, although in both cases further follow up data are lacking. One proceeded to regular IVIG (8 weekly) over several years, later treated with azathioprine, with reductions in seizure frequency and severity. Overall, however, only two of 16 patients became seizure free. Seizure freedom was attributed in both to AEDs and neither was treated with immunotherapy.

#### Cerebellar ataxia

Twenty-one patients had cerebellar ataxia (CA) which was progressive in all but two. The majority were clinically suspected or confirmed to have a neurodegenerative or inherited CA. Of these, two had suspected multisystem atrophy (MSA-C) and seventeen underwent genetic screening appropriate to the presentation (one had a spinocerebellar ataxia 17 mutation and one was heterozygous for a pathogenic SPG7 mutation). Eleven patients had a pure cerebellar syndrome. Eight patients had CA with one additional symptom or sign (CA with one of cognitive decline, myoclonus, opsoclonus-myoclonus, spasticity, palatal tremor, laryngospasm, pyramidal syndrome or headaches suggestive of familial hemiplegic migraine). Only three had suspected inflammatory/immune or paraneoplastic CA and five had uncertain diagnoses. Eleven of 19 patients (58%) had cerebellar atrophy on neuroradiology.

Despite this heterogeneity, 11 (52%) patients were treated with immunotherapy, six with first line therapy only (steroid monotherapy 2, IVIG monotherapy 2, IVIG with steroids 1 or IVIG with PLEX 1) and five second line, including azathioprine (2), cyclophosphamide (2) and mycophenolate (1). Only one patient (CA with opsoclonus-myoclonus and known RRMS with low positive GAD antibodies) had objective but partial benefit. Ten patients were not tried on immunotherapy, mostly due to low clinical suspicion for an autoimmune cause.

#### Mixed presentations

Twelve patients had a mixed phenotype, including 7 with SPS-SD plus either epilepsy only (3), CA only (2) or epilepsy and CA (2). Three patients with SPS-plus presentations had co-existent glycine receptor antibodies (one also with worsening diabetes in the context of neurological relapse). Of these, two had response to first line treatments, with one (also given rituximab) becoming negative for glycine receptor antibodies. The third was treated with steroids, PLEX (two cycles) and IVIG but showed no improvement. Of the other patients with ‘SPS-plus’, three had type 1 diabetes or LADA, with sequential onset of temporal lobe epilepsy then stiffness, and one had CA then stiffness, all showing modest improvement with IVIG.

Five patients had epilepsy plus CA. Of these two had confirmed genetic diagnoses, including one with xeroderma pigmentosum who showed modest improvement in SARA score with IVIG and one with autosomal recessive spastic paraplegia type 21 (not treated). One patient had clear separation of events with longstanding stable focal epilepsy since adolescence and then CA onset in the ninth decade (not treated with immunotherapy). The final two had a complex neurological syndrome in the 5th and 6th decade including seizures, CA, and brainstem symptoms including visual disturbance, diplopia and ‘dizziness’, one also with severe neurocognitive decline over 10 years. Of these, one (without neurocognitive decline) had very good improvement of CA with cyclophosphamide and the other (with neurocognitive decline) had no clear response to mycophenolate (tried late, 10 years after onset).

#### Other neurological disorders

Seventy-two patients had one or more neurological conditions other than a classical syndrome. The reasons for testing GAD antibodies included investigation of stiffness/abnormal tone or possible SPS-SD, paraneoplastic screens, or by endocrine specialists for non-neurological indications. The eventual diagnoses were very broad (Fig. 1). Of the 13 patients with values > 10,000 IU/mL, only five were treated. Of these, 2/5 (both values > 1,000,000 IU/mL) had typical autoimmune myasthenia gravis and improved. The other three patients were initially treated for possible SPS-SD. Of these, one was treated with six courses IVIG and one with two courses IVIG, one cycle PLEX and steroids, prior to revision of diagnoses to motor neuron disease and a complex neurodegenerative disease respectively. Both patients had initial reported but then non-sustained improvement with immunotherapy. The third patient experienced a deterioration in symptoms including loss of independent walking following a single cycle PLEX and the diagnosis was revised to inherited spastic paraparesis.

### Factors influencing treatment responses

OCBs and/or intrathecal antibody synthesis are often considered an indicator of an individual who may benefit from immunotherapies. Treating closer to onset of disease may be better than treating late chronic conditions. Additionally, very high GAD antibody values are usually considered more likely associated with an autoimmune neurological condition. Although numbers were small in our cohort, immunotherapy responses did not associate with duration of disease before treatment (using < or > 1year from onset), serum GAD-Ab value (using 10,000 IU/mL as a cut off), presence of unmatched oligoclonal bands in CSF or intrathecal synthesis of GAD-Abs (Table 3**, **Fig. 3c). Instead, treatment response associated best with the clinical syndrome.

**Table 3:** Factors associated with treatment response

Total number	No response	Partial response	Good response	Any response v no response	
SPS-SD, *n* = 19	4	10	5	15 v 4	*p* < 0.005*
Limbic encephalitis, *n* = 8	1	2	5	7 v 1	
Epilepsy, *n* = 4	2	1	1	2 v 2	
Ataxia, *n* = 9	8	1	0	1 v 8	
Mixed, *n* = 10	5	3	2	5 v 5	
Diabetes, *n* = 18	7	9	2	11 v 7	NS
No diabetes, *n* = 32	13	8	11	19 v 13	
Serum titer > 10,000 IU/mL, *n* = 33	12	13	8	21 v 12	NS
Serum titer < 10,000 IU/mL, *n* = 13	6	3	4	7 v 6	
Unmatched OCB, *n* = 19	8	8	3	11 v 8	NS
Matched or negative OCB, *n* = 13	4	5	4	9 v 4	
ITS, *n* = 8	4	3	1	4 v 4	NS
No ITS, *n* = 8	4	1	3	4 v 4	
OCB + ITS, *n* = 5	3	2	0	2 v 3	
Time from onset to treatment < 1 year, *n* = 24	7	9	8	17 v 7	NS
Time from onset to treatment > 1 year, *n* = 23	12	7	4	11 v 12	

Data refers to patients with available follow up data and deemed to have had an adequate trial of immunotherapy. Significance of presence/absence of diabetes, value > 10,000 IU/mL or < 10,000 IU/mL, presence or absence of unmatched oligoclonal bands (OCBs), presence or absence of intrathecal synthesis (ITS), and time of onset to treatment < 1year or > 1year in relation to treatment response determined by Chi-square test for trend* or fisher exact tests

ITS: intrathecal synthesis; OCB: oligoclonal bands; SPS-SD stiff person syndrome spectrum disorders

### Side effects to immunomodulatory therapies (whole cohort)

Assessing morbidity related to immunomodulatory therapy in this retrospective cohort was challenging. Most complications occurred with PLEX, including three deep vein thromboses associated with femoral Vascath, one with significant hematoma. Of these, two (one SPS-SD, one encephalitis) had response to immunotherapy. Two patients (one epilepsy, one mixed) with IDDM had worsening hyperglycemia with steroids without benefit from treatment. One patient had life-threatening sepsis related to a Portacath device placed for rituximab but had good response to immunotherapy. Azathioprine was stopped in three patients (one SPS-SD, two epilepsy) for neutropenia, (benign) skin lesions and one for side effects not specified. One patient (mixed) did not tolerate MMF but responded to cyclophosphamide.

## Discussion

The clinical relevance of GAD-Abs in neurology is challenging. The ELISA assay commonly used was established for diagnosis of IDDM rather than to measure the high levels of GAD-Abs thought to be important in SPS-SDs. GAD-Abs have also been found in patients with CA, encephalitis and epilepsy, or mixed neurological syndromes and there is a need to provide guidelines for interpretation of the values and their clinical relevance in practice. We reviewed 335 consecutive positive GAD-Abs patients from 2012 to 2020. Of those with neurological syndromes, only 57% had SPS-SD or a condition previously described with GAD-Abs; the remainder represented a heterogeneous group with inflammatory and non-inflammatory disorders of the central and peripheral nervous system, or psychiatric presentations. Concentrating on the 57%, a cut-off between low and high GAD-Abs of 10,000 IU/mL was neither sensitive nor specific for distinguishing GAD-Ab neurological disorders.

Patients with SPS-SD (or mixed SPS syndromes) often had high GAD-Abs, accompanied by intrathecal GAD-Ab synthesis and/or oligoclonal bands, and typically responded to sustained immunotherapies. However, 9/23 SPS-SD cases had low positive GAD-Abs and 4/7 of these also made partial or good immunotherapy responses. Patients with an autoimmune encephalitis also tended to make a good treatment responses, but patients with isolated epilepsy were both less likely to be treated (low suspicion of autoimmune etiology) and less likely to respond to treatment. Discouraging results were also found in those with CA, with a partial response in only one of eleven treated CA patients (with the lowest serum value of those treated). Though conclusions here are limited by numbers and heterogeneity of treatment regimens used, overall in this cohort, the presence of very high GAD-Abs, oligoclonal bands or intrathecal synthesis did not appear to influence immunotherapy response.

Indeed the relevance of “high” value GAD-Ab values is not at all clear. GAD-Abs are not required for the diagnosis of the classical GAD antibody-associated syndromes and may be absent in more than 50% [14, 15]. Similarly, as seen here, some patients with classical syndromes have low values [16]. The issue has not been helped by the fact that many of the earlier studies reporting cerebellar ataxia with high GAD-Abs were performed with a different GAD-Ab assay, reported in U/mL rather than IU/mL as used more recently [8, 11]. Most laboratories and clinicians are not aware of the 25-fold difference in values between these assay types whereby 2000 U/mL would be 50,000 IU/mL with the current assay. High variability and lack of specificity of GAD-Abs for neurological disease, has also previously been highlighted using the radioimmunoprecipitation assay [17].

We found higher serum GAD-Ab values in patients with classical GAD-Ab syndromes and co-existing IDDM. Thus it may be that the concomitant autoimmune processes (non-neuronal autoantibodies were common) affect the serum antibody value, and it implies that high GAD-Ab values in patients with co-existing diabetes should not imply an immunotherapy responsive neurological disorder. What makes GAD so immunogenic compared to other brain antigens in these specific settings remains unclear.

Probable intrathecal Ab synthesis was demonstrated in SPS-SD, mixed syndromes and LE, in keeping with previous studies [18, 19] With high values of antibodies in the serum, there will inevitably be measurable antibodies passively transferred to the CSF; only if the CSF value is higher than expected from transfer across the blood brain barrier can intrathecal synthesis be identified (assuming the absence of a dysfunctional blood brain barrier). The CSF values in the few epilepsy patients tested indicated no significant intrathecal synthesis, and most cases did not have any clear autoimmune basis. In some cases, pathogenic autoantibodies to more probable targets are recognized, including the GABA_A_ or GABA_B_ receptors in encephalitis; these patients tend to respond well to immunotherapies, but no neuronal surface target antigen has been identified in the majority of GAD-Ab epilepsy patients [20]. Indeed, the pathogenic role of GAD antibodies has always been in doubt [21]. Instead GAD-Abs may be secondary to irreversible T cell-mediated or other damage leading to release of intracellular GAD, perhaps occurring in patients with or at high risk of autoimmunity, leading to autoantibody generation [22–24]. The role of T cells causing neuronal injury in GAD-Ab associated temporal lobe epilepsy has recently been demonstrated [25].

Whilst some CA patients were suspected to have an autoimmune basis (including some in the ‘mixed’ group), in most, the progressive course, older age and in some cases family history suggested inherited or neurodegenerative processes. In fact, two patients had confirmed genetic causes, one with GAD-Ab value 178,992 IU/mL, perhaps driven by co-existing IDDM. The question then arises why cerebellar degeneration is more associated with GAD-Ab synthesis than other neurodegenerative disease or brain injury. Intrathecal GAD-Ab synthesis seen in four CA patients suggests an immune response in the CNS compartment, possibly as GAD is highly expressed in the cerebellum [8, 26]. In addition, one cannot ignore a bias towards investigating patients with CA for GAD-Ab because of the recognized association and in the hope of a beneficial treatment response. The OND group also included 14 patients with high GAD-Abs but no intrathecal synthesis in 3/4 available. The exception was a patient initially suspected to have SPS-SD but no response to immunotherapies and subsequently given a neurodegenerative diagnosis.

This is a ‘real world’ study from a center with extensive clinical and laboratory diagnostic experience, but our cohort was dependent on requester/tertiary center preferences. Other study limitations include retrospective design, limited CSF data requiring use of a surrogate for intrathecal synthesis and immunotherapies given or not dependent mainly on the referring neurologist, often with variable follow up. Unfortunately, duration of disease, GAD-Ab values, intrathecal synthesis or oligoclonal bands were not clearly helpful in determining treatment responses. However, it appears that patients with SPS-SD (including when part of mixed syndromes) or encephalitis are likely to respond to immunotherapies at least partially, whereas this was not the case for the epilepsy or CA patients. More specific biomarkers, perhaps potentially pathogenic antibodies appropriate for the clinical syndromes, could greatly improve the accuracy by which treatment-responsive patients can be identified. In addition, multicenter prospective randomized-controlled trials using standardized treatment approaches and robust outcome measures should be undertaken.
